# Natural History of Untreated Optic Neuritis Associated With Mild COVID-19 Infection

**DOI:** 10.7759/cureus.42168

**Published:** 2023-07-19

**Authors:** Eileen Javidi, Samir Touma, Fares Antaki, Daniela Toffoli

**Affiliations:** 1 Department of Ophthalmology, Université de Montréal, Montreal, CAN; 2 Department of Ophthalmology, Centre Hospitalier de l’Université de Montréal (CHUM), Montreal, CAN

**Keywords:** neuro-ophthalmic complications of covid-19, demyelinating diseases, sars-cov-2, covid-19, optic neuritis

## Abstract

This article describes a case of untreated optic neuritis occurring in the setting of coronavirus disease 2019 (COVID-19) infection and provides new insights into the natural history of this condition. A 29-year-old male patient with no known ocular or systemic disease presented with pain on extraocular movements and sudden loss of vision in the inferior visual field affecting the right eye. He had tested positive for COVID-19 six days prior after experiencing mild upper respiratory symptoms. On examination, visual acuity was 20/20, and color vision was normal. A relative afferent pupillary defect was observed in the right eye. Fundoscopy revealed mild optic disc edema in the same eye. Optical coherence tomography showed increased retinal nerve fiber layer thickness of the right optic nerve head and visual field testing revealed an inferonasal defect. Extensive laboratory and imaging investigations failed to reveal an underlying etiology, supporting a diagnosis of COVID-19-associated optic neuritis. The patient improved spontaneously without treatment. At the five-month follow-up, minor optic atrophy and a small residual visual field defect remained.

## Introduction

Coronavirus disease 2019 (COVID-19) has been spreading worldwide since the first case was identified in December 2019 [1]. This disease causes infections affecting mainly the respiratory system with varying severity, from mild to life-threatening [2]. It has also been reported to have extra-pulmonary manifestations such as ophthalmic involvement [3]. Documented cases of COVID-19 neuro-ophthalmologic complications have been varied and include afferent complications such as optic neuropathies and papilledema, as well as efferent complications such as cranial neuropathies, Miller Fisher syndrome, and nystagmus, among others [4].

Optic neuritis (ON) refers to inflammation of the optic nerve and is typically characterized by painful vision loss accompanied by an afferent pupillary defect. The disease requires a thorough evaluation, including neuroimaging and laboratory tests, to consider the potential underlying causes which include autoimmunity, demyelination, infection, and granulomatous disease. This approach is important because the prognostic variance between etiologies is very significant. For example, cases associated with multiple sclerosis (MS) are often self-limited with a high chance of recovery, while those associated with neuromyelitis optica spectrum disorder (NMOSD) are typically severe, showing more limited visual recovery [5].

ON associated with COVID-19 is thought to more likely occur due to “triggered autoimmunity and immunologic upregulation” [4] rather than direct viral invasion and may present in the setting of viral infections of varying severity, ranging from a complete absence of respiratory symptoms [6] to mild infection [7] and severe complications requiring ICU hospitalization [8]. Such cases were managed with intravenous corticosteroids followed by tapering oral doses, with a favorable response in the majority of cases [6,8]. To the best of our knowledge, no previous reports describe the natural history of untreated ON in the setting of COVID-19 infection. This report expands our understanding of neuro-ophthalmic complications of COVID-19 by providing new insight into the underlying pathophysiology of COVID-19-associated ON, through its clinical similarities with MS-associated demyelinating ON.

## Case presentation

A 29-year-old man with no relevant medical history was referred to our tertiary ophthalmology clinic for suspected ON. The patient had noticed sudden loss of vision in the inferior visual field of his right eye, associated with pain with extraocular movements. He had no other neurological symptoms. Six days prior, he had tested positive for severe acute respiratory syndrome coronavirus 2 (SARS-CoV-2) on nasopharyngeal swab rapid antigen test after experiencing mild symptoms of headache and sore throat. He had previously received three doses of the BNT162b2 mRNA COVID-19 vaccine (Pfizer-BioNTech).

**Figure 1:**
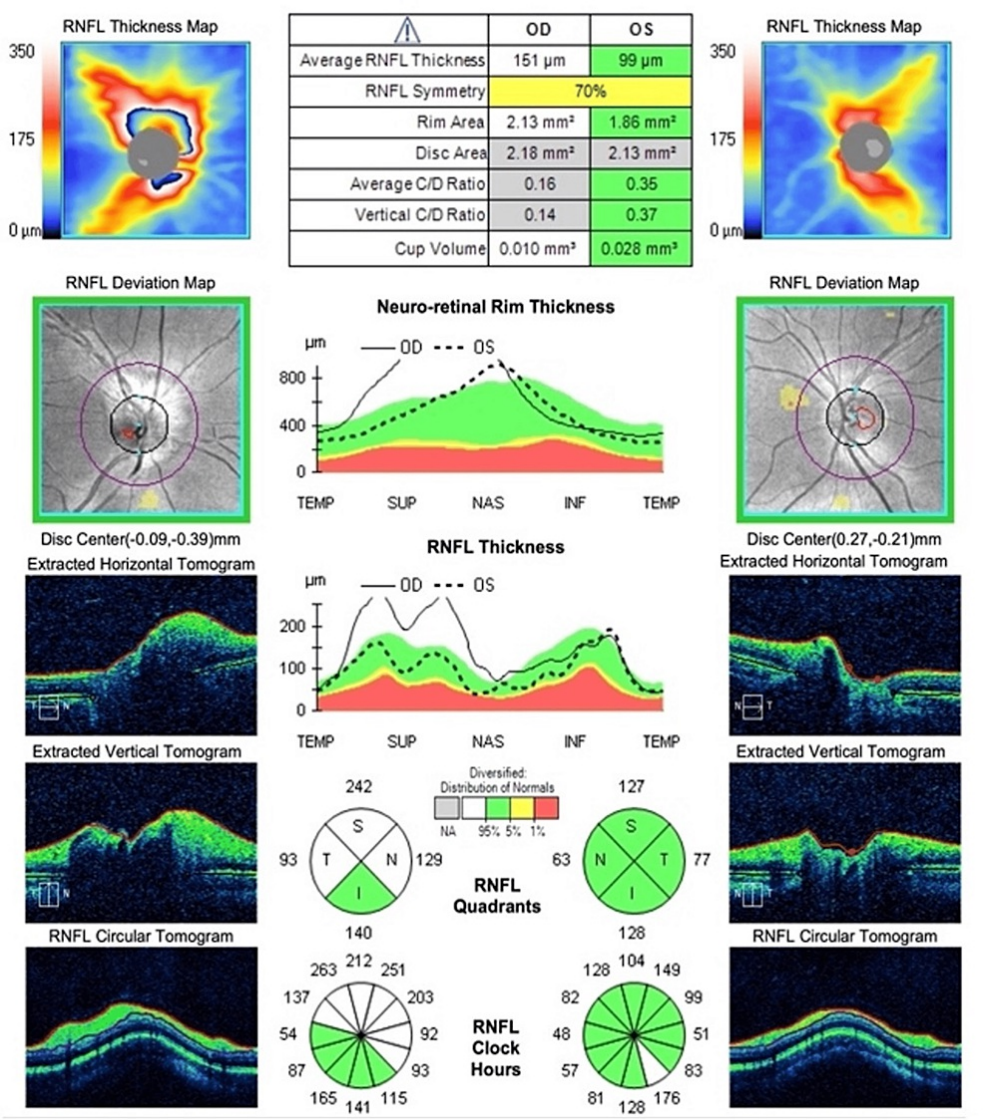
Optical coherence tomography of the right (shown on left) and left (shown on right) optic nerve head (Cirrus; Carl Zeiss Meditec, Inc. Dublin, CA, USA) at the initial presentation, showing increased RNFL thickness in the superior, nasal, and temporal quadrants OD. RNFL: retinal nerve fiber layer; OD: right eye; OS: left eye

**Figure 2:**
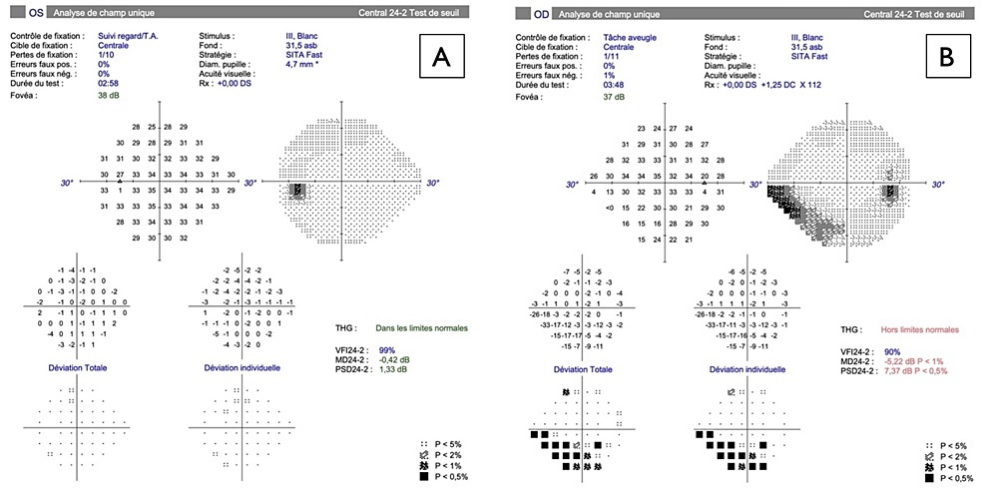
Visual field testing of OD and OS on 24-2 SITA Fast Humphrey Field Analyzer (HFA3; Carl Zeiss Meditec) at the initial presentation, showing A) Normal visual field OS, and B) Inferonasal defect OD. OD: right eye; OS: left eye

Uncorrected visual acuity was 20/20 in both eyes, and intraocular pressure was 18 and 20 mmHg in the right and left eyes. Color vision was normal in both eyes (measured using Hardy, Rand and Rittler (HRR) plates). Extraocular movements were full, but painful in his right eye. Pupils were round and isocoric. There was a right relative afferent pupillary defect. Examination of the remaining cranial nerves revealed no abnormalities. Anterior segment examination was normal in both eyes. Fundus examination of the right eye revealed a normal-coloured optic disc with superior edema, and a normal macula and periphery. The left fundus was normal. Optical coherence tomography (OCT) of the right optic nerve head showed thickening of the retinal nerve fiber layer (RNFL) in the superior, nasal, and temporal quadrants (Figure 1), corresponding with an inferonasal defect revealed on visual field (Figure 2).

The patient was diagnosed with unilateral ON associated with mild COVID-19. Other diagnoses such as non-arteritis ischemic optic neuropathy (NAION) were considered less probable by the authors. Of note, the patient had a contralateral estimated cup/disc ratio of 0.35 on OCT as shown in Figure 1; in a study by Beck et al. involving 51 patients with NAION, it was found that “forty-six (91%) of the involved eyes and 37 (73%) of the fellow eyes had no physiologic cup. None of the involved eyes and only three of the fellow eyes had a cup/disc ratio as great as 0.3” [9]. Given this patient’s clinical features (absence of a contralateral crowded disc, absence of flame-shaped hemorrhages on fundus examination), young age, and lack of potential risk factors for NAION, the diagnosis of anterior optic neuritis was considered far more probable. Treatment with a three-day course of intravenous methylprednisolone followed by oral prednisone taper was considered, in a fashion similar to management of MS-associated ON [10]. However, given very good visual function, a decision between the patient, neurology consultants and ophthalmology was made for close observation without treatment. An extensive workup was performed to search for demyelinating, infectious, immune-mediated, and other etiologies of ON (Table 1). Most importantly, magnetic resonance imaging of the brain and orbits was unremarkable, showing no demyelinating or infiltrating lesions, and anti-aquaporin 4 (AQP4) antibodies and anti-myelin oligodendrocyte glycoprotein (MOG) antibodies were negative. However, a lumbar puncture and cerebrospinal fluid (CSF) analysis were not performed, given the mild visual dysfunction and absence of atypical signs and symptoms.

**Table 1:** Laboratory investigations and imaging studies conducted CRP: C-reactive protein; ESR: erythrocyte sedimentation rate; ACE: angiotensin-converting enzyme; HIV: human immunodeficiency virus; EIA: enzyme immunoassay; TB: tuberculosis; AQP4: aquaporin 4; MOG: myelin oligodendrocyte glycoprotein; MRI: magnetic resonance imaging

Investigation	Result	Interpretation
Blood tests		
CRP	<5 mg/L; normal < 10	Normal
ESR	2 mm/H; normal 2-28	Normal
ACE	46 U/L; normal 10-83	Normal
Lysozyme	9.22 ng/mL; normal 5.00-10.00	Normal
HIV antibodies and p24 antigen	Negative	-
EIA-Syphilis	Negative	-
Quantiferon-TB Gold	Negative	-
Anti-AQP4 antibodies	Negative	-
Anti-MOG antibodies	Negative	-
Imaging		
Chest X-Ray	Normal	-
Cerebral and orbital MRI with gadolinium	Normal without optic nerve enhancement or white matter lesions	-

**Figure 3:**
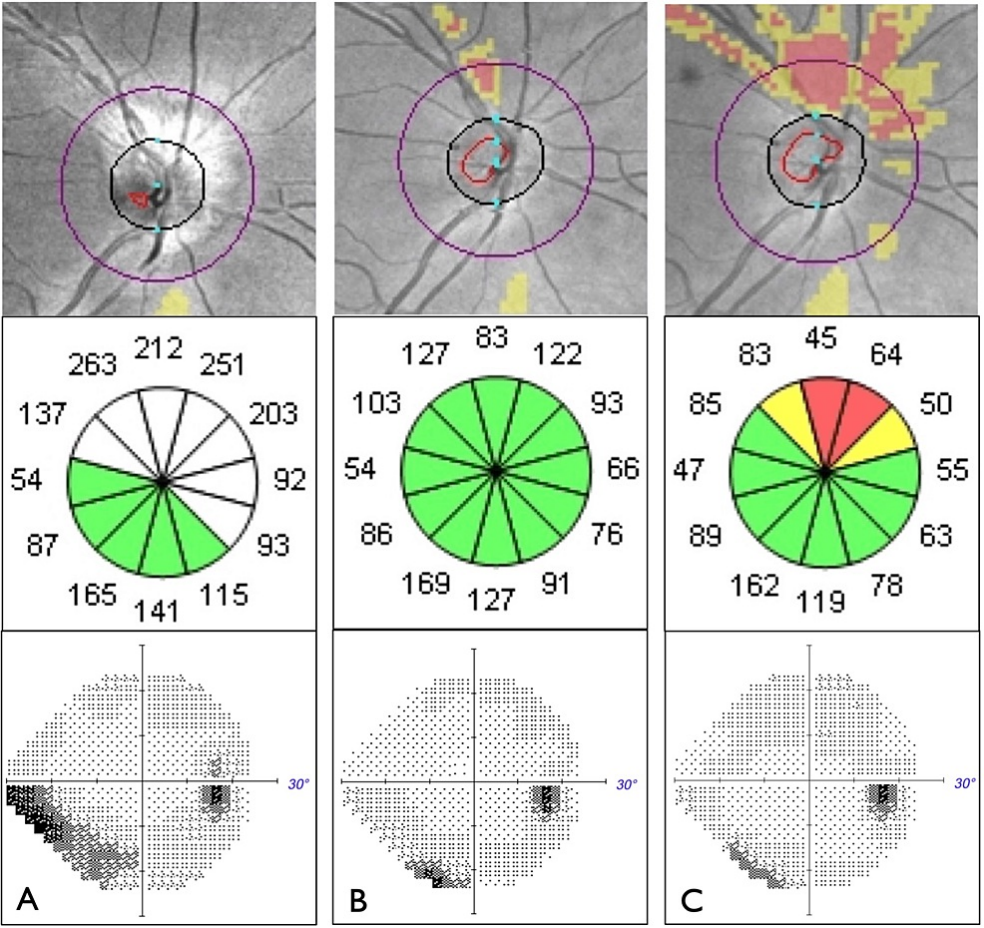
RNFL deviation map (top) and RNFL clock hours (middle) on OCT with corresponding right VF testing (bottom) throughout follow-up, showing A) ON edema with corresponding inferonasal VF defect at initial presentation (mean deviation -5.22 dB), B) decreasing ON edema with improved VF defect at one-month follow-up (mean deviation -2.37 dB), and C) slight residual ON atrophy with a small VF defect at five-month follow-up (mean deviation -2.80 dB). RNFL: retinal nerve fiber layer; OCT: optical coherence tomography, ON: optic nerve; VF: visual field

Ocular pain resolved spontaneously within two weeks and subjective vision improved at every visit. Vision remained 20/20 without dyschromatopsia. At the one-month follow-up, the optic nerve edema was no longer visible on fundoscopy. At that time, OCT RNFL thickness measurements had decreased to within normal limits (Figure 3B). At five months, disc pallor was visible on fundoscopy and corresponding RNFL thinning was seen on OCT (Figure 3C). Macular and ganglion cell complex thickness remained normal in both eyes throughout follow-up.

## Discussion

Although COVID-19 is primarily a disease of the respiratory system, it has also been reported to cause a wide range of central nervous system (CNS) manifestations, many of which are associated with demyelination such as encephalitis (including acute disseminated encephalomyelitis), longitudinally extensive transverse myelitis and optic neuritis [11]. While these remain rare in comparison to respiratory symptoms, the overall number of patients presenting with demyelinating events post-COVID-19 could become increasingly significant due to the continuing spread of the virus, even in the post-pandemic era. Therefore, it is important to better understand the neurological and neuro-ophthalmic manifestations of COVID-19 [12].

The pathogenesis of COVID-19 neurological complications has yet to be fully elucidated, but several theories have been proposed. The COVID-19 virus uses the angiotensin-converting enzyme-2 (ACE2) receptor to enter cells, which is highly expressed in the CNS and retinal cells. It has therefore been hypothesized that neurotropism occurs due to the virus’s affinity for the ACE2 receptor [13]. Furthermore, COVID-19 can cross the blood-brain barrier to create a cytokine storm and a proinflammatory state, which may contribute to tissue damage in the CNS and immunologic dysfunction [11]. Another mechanism by which COVID-19 results in demyelination may also include molecular mimicry. COVID-19 antigens elicit an abnormal immune response, resulting in the production of autoantibodies targeting CNS myelin proteins [14,15].

In the current context, we believe that patients with new onset of ON should be asked about a recent history of COVID-19. Even if an association is suspected, extensive investigations remain essential in order to identify a potential underlying autoimmune disease that may have been triggered by the virus [4,14], as the clinical outcomes and appropriate management vary considerably between diseases. ON in the setting of NMOSD and MOG antibody disease (MOGAD) is typically more severe and at high risk for relapse, compared to MS-associated ON, which has a favorable prognosis [5]. This distinction is important because there is growing literature about NMOSD and MOGAD in patients with ON associated with COVID-19. A recent literature review of 18 cases of COVID-19-associated CNS inflammatory demyelinating diseases found the most common to be NMOSD (39%), followed by MOGAD (28%) and MS (22%), while isolated ON comprised 11% of cases [15]. The latter was the most probable entity in our patient. His clinical presentation was not suggestive of NMOSD or MOGAD, his cerebral imaging was normal, and the anti-MOG and anti-APQ4 antibodies were negative.

Our case was not treated with corticosteroids or other medical therapies and therefore sheds light on the natural history of COVID-19-associated ON, revealing a clinical course that appears similar to that of clinically isolated syndrome or MS-related ON. Initial presentation with acute unilateral decrease in visual acuity progressing over one to two weeks followed by spontaneous recovery is typical of clinically isolated syndrome or MS-related ON [5]. Pain with eye movements, reported in 92% [10] of patients from the ON Treatment Trial (ONTT), was also predominant in our patient. However, his fundoscopic examination revealed optic disc edema, which is less common in clinically isolated syndrome or MS-associated ON but can be present in 35% of cases [10]. Additionally, while diffuse or central loss are the most common types of visual field loss in MS-related ON, a variety of other visual field defects have been documented, such as arcuate deficits as seen in our patient [16]. ON in clinically isolated syndrome or MS is due to immune-mediated demyelination of the optic nerve; as currently hypothesized in the literature, COVID-19 may also induce a host response against myelin in some patients [4,14]. Viral-associated demyelination has also been reported with other coronavirus subtypes as well as Epstein-Barr virus [15]. The prognostic implications of this potential correlation are unknown, and it remains unclear whether our patient might be at risk for future episodes.

The current standard for treatment of acute typical ON is high-dose IV methylprednisolone (1 kg/day for three days) followed by oral prednisolone taper over 11 days. Therapy allows for an accelerated rate of visual recovery but does not modify long-term visual outcomes at one-year follow-up and beyond. Recommendations based on the ONTT differ depending on the severity of visual loss; treatment with IV methylprednisolone followed by oral prednisolone does not seem to be beneficial in patients with visual acuity of 20/40 or better and minimal visual field defects, but should be considered in patients with vision worse than 20/40 [10]. Additionally, corticosteroid treatment is warranted in the acute phase for atypical cases of ON until investigations are completed, and treatment can be tailored towards the underlying etiology [17]. Alternate etiologies should be suspected when atypical features are present, which may include bilaterality, no light perception, vision loss progressing over more than two weeks, lack of recovery, painlessness, anterior segment inflammation, fundus anomalies (severe disc edema, peripapillary hemorrhages, macular exudates), age below 18 or above 50, and male sex [18]. In our case, the potential benefits of slightly accelerated visual recovery were weighed against the uncertainty regarding etiology and the patient’s desire to avoid invasive therapy. Considering the good visual function and spontaneous recovery, observation was favored.

## Conclusions

We report the case of an otherwise healthy 29-year-old male patient who developed unilateral ON six days after COVID-19 infection; notably, anti-APQ4 and anti-MOG antibodies were negative and cerebral imaging was unremarkable. Although there have been previous reports of isolated ON associated with COVID-19, this paper is, to our knowledge, the first describing the natural history of this disease when left untreated. Our case shows a clinical portrait comparable in presentation and disease progression to that of classical clinically isolated syndrome and MS-associated ON with good visual outcome. Indeed, virally associated demyelination may likely be causative in our patient, although it remains possible that there was merely a temporal association between our patient’s COVID-19 diagnosis and his optic neuritis. Long-term follow-up of our patient is needed to establish whether such events are isolated or may recur. As the literature on the neuro-ophthalmic complications of COVID-19 continues to grow, further research will undoubtedly lead to better insight into the virus’s mechanisms of ocular injury.

## References

[1] Zhu N, Zhang D, Wang W (2020). A novel coronavirus from patients with pneumonia in China, 2019. N Engl J Med.

[2] Wu Z, McGoogan JM (2020). Characteristics of and important lessons from the coronavirus disease 2019 (COVID-19) outbreak in China: Summary of a report of 72 314 cases from the Chinese Center for Disease Control and Prevention. JAMA.

[3] Zhong Y, Wang K, Zhu Y, Lyu D, Yu Y, Li S, Yao K (2021). Ocular manifestations in COVID-19 patients: a systematic review and meta-analysis. Travel Med Infect Dis.

[4] Tisdale AK, Dinkin M, Chwalisz BK (2021). Afferent and efferent neuro-ophthalmic complications of coronavirus disease 19. J Neuroophthalmol.

[5] Bennett JL (2019). Optic neuritis. Continuum (Minneap Minn).

[6] Deane K, Sarfraz A, Sarfraz Z, Valentine D, Idowu AR, Sanchez V (2021). Unilateral optic neuritis associated with SARS-CoV-2 infection: a rare complication. Am J Case Rep.

[7] Duran M, Aykaç S (2023). Optic neuritis after COVID-19 infection: a case report. J Fr Ophtalmol.

[8] Azab MA, Hasaneen SF, Hanifa H, Azzam AY (2021). Optic neuritis post-COVID-19 infection. A case report with meta-analysis. Interdiscip Neurosurg.

[9] RW Beck, PJ Savino, MX Repka, NJ Schatz, RC Sergott (1984). Optic disc structure in anterior ischemic optic neuropathy. Ophthalmology.

[10] Beck RW, Cleary PA, Anderson MM Jr (1992). A randomized, controlled trial of corticosteroids in the treatment of acute optic neuritis. The Optic Neuritis Study Group. N Engl J Med.

[11] Ismail II, Salama S (2022). Association of CNS demyelination and COVID-19 infection: an updated systematic review. J Neurol.

[12] Ellul MA, Benjamin L, Singh B (2020). Neurological associations of COVID-19. Lancet Neurol.

[13] Lukiw WJ, Pogue A, Hill JM (2022). SARS-CoV-2 infectivity and neurological targets in the brain. Cell Mol Neurobiol.

[14] Zhou S, Jones-Lopez EC, Soneji DJ, Azevedo CJ, Patel VR (2020). Myelin oligodendrocyte glycoprotein antibody-associated optic neuritis and myelitis in COVID-19. J Neuroophthalmol.

[15] Feizi P, Sharma K, Pasham SR, Nirwan L, Joseph J, Jaiswal S, Sriwastava S (2022). Central nervous system (CNS) inflammatory demyelinating diseases (IDDs) associated with COVID-19: a case series and review. J Neuroimmunol.

[16] Keltner JL, Johnson CA, Cello KE, Dontchev M, Gal RL, Beck RW (2010). Visual field profile of optic neuritis: a final follow-up report from the optic neuritis treatment trial from baseline through 15 years. Arch Ophthalmol.

[17] Malik A, Ahmed M, Golnik K (2014). Treatment options for atypical optic neuritis. Indian J Ophthalmol.

[18] Abel A, McClelland C, Lee MS (2019). Critical review: typical and atypical optic neuritis. Surv Ophthalmol.

